# Suspected Urine-Induced Chemical Peritonitis Secondary to Invasive Bladder Cancer: A Case Report

**DOI:** 10.7759/cureus.90092

**Published:** 2025-08-14

**Authors:** Stanislaw Szymkiewicz

**Affiliations:** 1 Department of Urology, Janusz Korczak Provincial Specialist Hospital, Slupsk, POL

**Keywords:** advanced urothelial carcinoma, bladder cancer, bladder perforation, cystectomy, obstructive uropathy, tumor necrosis, uremic peritonitis

## Abstract

Chemical (urine-induced) peritonitis is a rare but potentially life-threatening complication in patients with advanced bladder cancer. It may result from sterile urinary leakage into the peritoneal cavity, typically through microscopic wall disruption or tumor necrosis. This condition can mimic perforation or bacterial peritonitis, posing a significant diagnostic challenge. We describe the case of a 78-year-old male with de novo high-grade urothelial carcinoma, presenting with persistent gross hematuria, bilateral hydronephrosis, and severe anemia. Despite initial tumor resection and nephrostomy placement, the patient’s condition deteriorated with signs of peritoneal irritation and systemic inflammation. Emergency cystoprostatectomy revealed extensive tumor necrosis and inflammatory peritoneal fluid, but no macroscopic bladder perforation. Histopathology confirmed muscle-invasive high-grade urothelial carcinoma (pT2b) with necrosis and angioinvasion. This report illustrates a diagnostic and therapeutic challenge in the management of advanced bladder cancer complicated by suspected sterile chemical peritonitis, emphasizing the importance of early source control in critically ill patients.

## Introduction

Bladder cancer is the most common malignancy of the urinary tract, typically presenting as painless hematuria and diagnosed at an early, non-muscle-invasive stage [1,2]. However, in advanced stages, tumor progression may lead to severe complications including obstructive uropathy, hemorrhagic cystitis, tumor necrosis, and, in rare cases, peritoneal inflammation possibly related to sterile urinary leakage [3,4]. Sterile chemical peritonitis secondary to bladder cancer is an uncommon and underrecognized clinical entity. It may result from extravasation of sterile urine into the peritoneal cavity, either through microscopic wall disruption, tumor infiltration, or necrosis. The clinical picture may mimic spontaneous bacterial peritonitis (SBP) or visceral perforation, making prompt diagnosis and appropriate management difficult [5].

To date, only a few case reports have described this phenomenon in the setting of urothelial carcinoma. Differentiating sterile peritonitis due to chemical irritation from infectious or malignant etiologies (e.g., peritonitis carcinomatosa) remains challenging without direct biochemical analysis of peritoneal fluid. In this report, we present the case of an elderly patient with de novo muscle-invasive urothelial carcinoma complicated by bilateral hydronephrosis, persistent inflammation, and suspected sterile chemical peritonitis, which was successfully managed with emergency radical cystoprostatectomy and urinary diversion.

## Case presentation

A 78-year-old male was referred from the Emergency Department to the urology ward with a three-month history of persistent gross hematuria, progressive anemia, bilateral hydronephrosis, and general clinical decline. He also reported moderate dysuria, although the severity of hematuria clinically overshadowed this symptom. Additionally, he described reduced appetite and unintentional weight loss in the weeks preceding admission; however, physical examination revealed preserved muscle mass and no signs of clinically significant malnutrition. Upon admission, the patient was hemodynamically stable, with a low-grade fever of 37.4 °C. The abdominal examination revealed a soft, non-tender abdomen with no guarding or rebound tenderness. Blumberg’s and Goldflam’s signs were negative, and bowel sounds were normal, effectively ruling out acute surgical or renal pathology at that time.

Initial laboratory evaluation demonstrated profound anemia (hemoglobin: 4.8 g/dL), elevated inflammatory markers (C-reactive protein (CRP): 26.9 mg/L), and impaired renal function (creatinine: 2.76 mg/dL). Urinalysis confirmed macroscopic hematuria, accompanied by leukocyturia and bacteriuria. Urine culture grew Providencia alcalifaciens (extended-spectrum beta-lactamase (ESBL)-negative). Key laboratory values across the clinical course are summarized in Table 1. The patient’s past surgical history included duodenal ulcer repair and appendectomy in the 1990s. There was no prior history of urological disease or instrumentation.

**Table 1:** Summary of laboratory parameters at different time points CRP: C-reactive protein; ESBL: extended-spectrum beta-lactamase; HPF: high-power field (microscopy); WBC: white blood cells

Parameter	Reference range	On admission	Pre-cystectomy	Pre-discharge
Hemoglobin, g/dL	13.5–17.5 (male)	4.8	9.7	10.8
CRP, mg/L	<5	26.9	297	88
Procalcitonin, ng/mL	<0.05	0.29	2.5	0.23
Creatinine, mg/dL	0.7–1.2	2.76	2.4	1.71
WBC, ×10⁹/L	4.0–10.0	8.06	16.11	10.56
Urinalysis	—	Gross hematuria, leukocyturia (50–70 WBC/HPF), bacteria	N/A	Microscopic hematuria only
Urine culture	—	Providencia alcalifaciens (ESBL-negative)	N/A	Sterile

**Figure 1:**
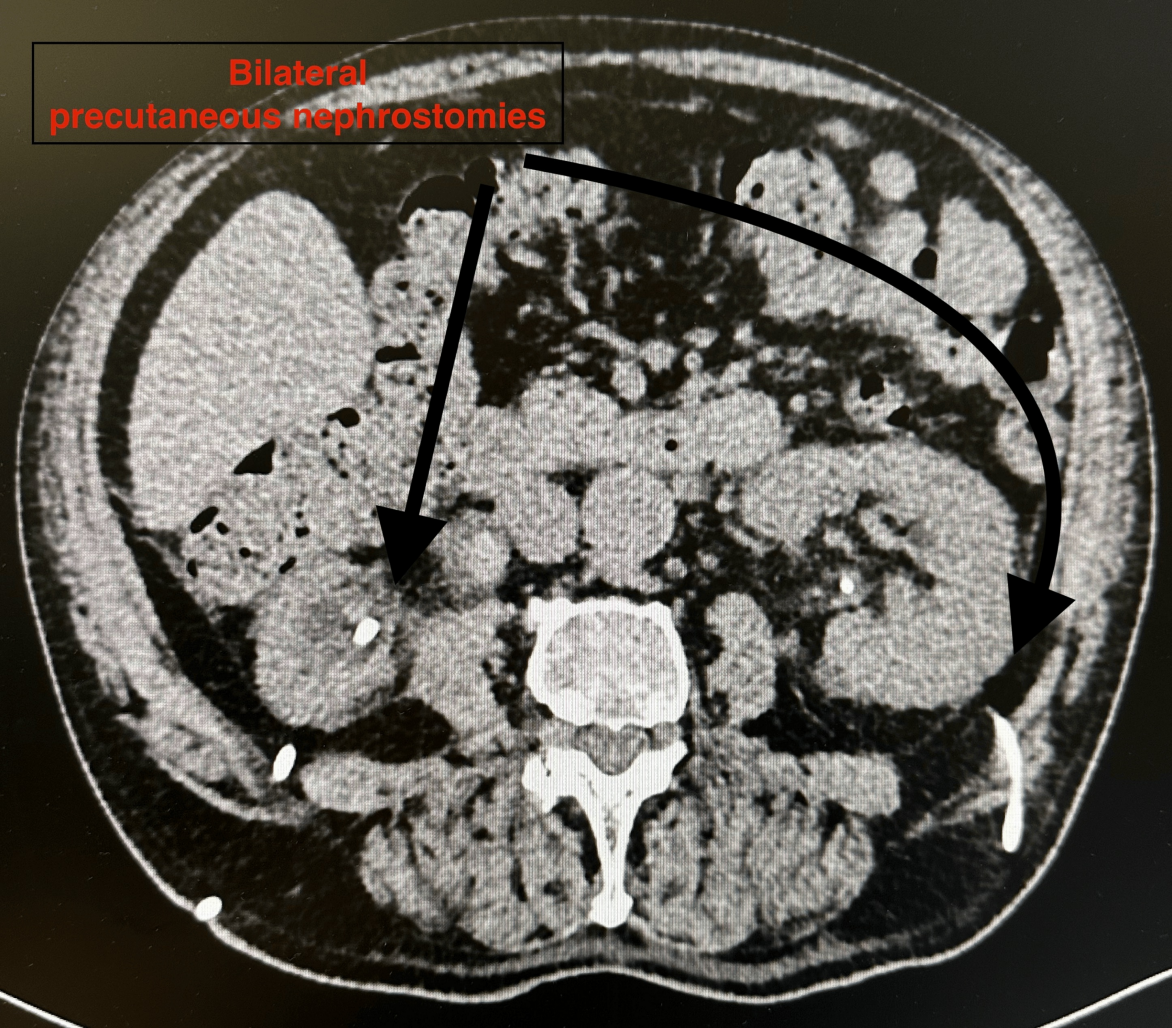
Axial CT image showing bilateral percutaneous nephrostomy tubes (black arrows) placed for relief of obstructive uropathy Moderate bilateral hydronephrosis is also visible CT: computed tomography

**Figure 2:**
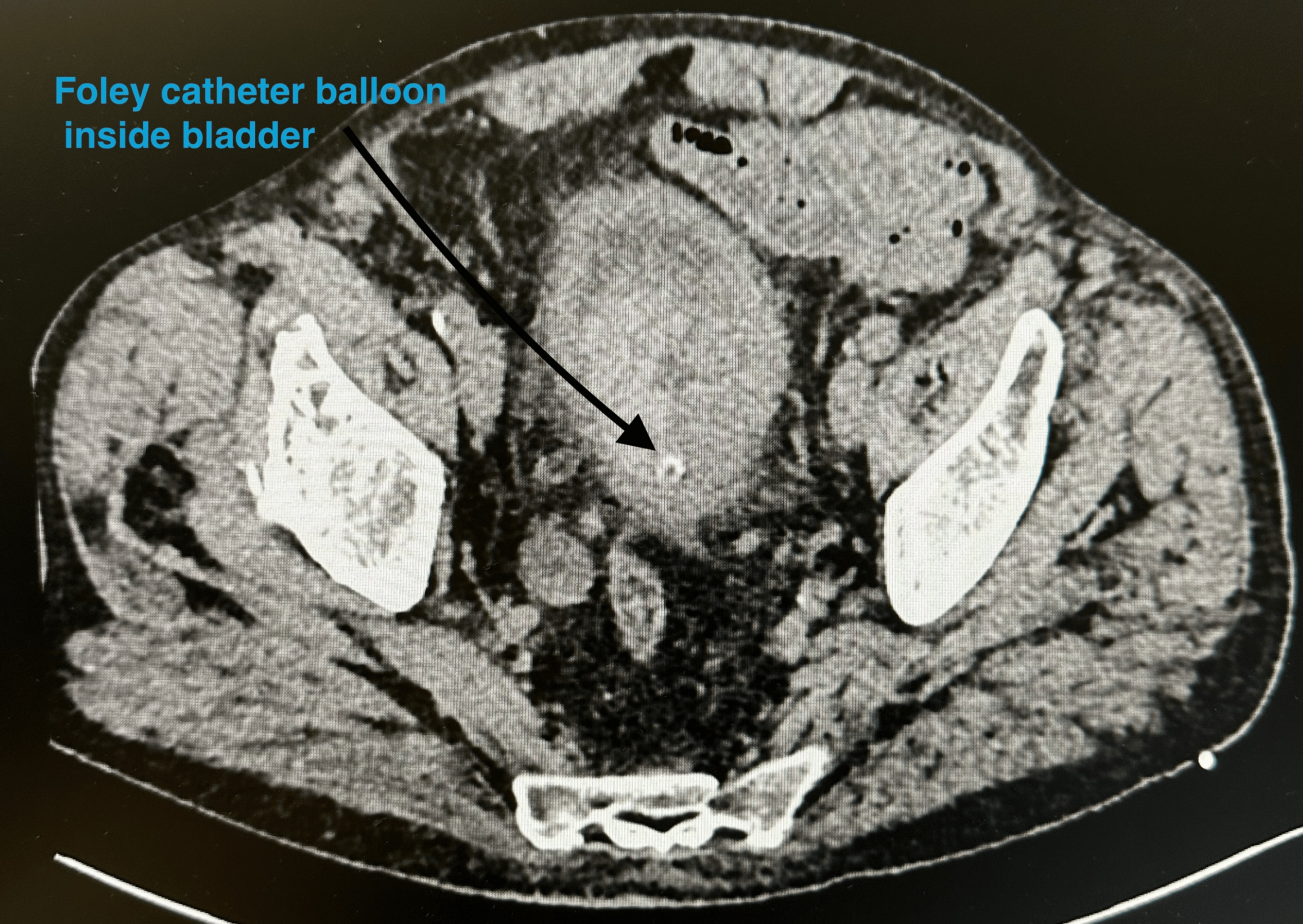
Axial CT scan at the level of the pelvis demonstrating a Foley catheter balloon inside the urinary bladder (black arrow) The bladder is enlarged and contains heterogeneous material suspicious for tumor debris or clot CT: computed tomography

Initial abdominal ultrasound and non-contrast CT revealed bilateral hydronephrosis and a large, irregular bladder mass measuring approximately 74 × 58 mm, with features suggestive of extensive local infiltration [2]. Although sagittal reconstructions were not available, axial CT slices demonstrated a heterogeneous mass consistent with a necrotic tumor and signs of bladder wall involvement, without intraperitoneal free air (Figures 1, 2). After initial stabilization, diagnostic cystoscopy revealed a large, friable tumor occupying the entire bladder diverticulum and extending toward the right posterior and lateral walls. The patient subsequently underwent transurethral resection of the bladder tumor (TURBT), during which approximately 90% of the visible lesion was successfully resected [2]. Intraoperatively, a microscopic perforation of the bladder wall was suspected, although no macroscopic defect was noted [3]. To relieve urinary obstruction, bilateral percutaneous nephrostomy tubes were inserted under ultrasound guidance.

Empirical broad-spectrum antibiotic therapy with piperacillin-tazobactam and levofloxacin was initiated, later escalated to meropenem following microbiological susceptibility results. Supportive treatment included tranexamic acid, etamsylate, proton pump inhibitors, low-molecular-weight heparin, probiotics, and careful fluid balance monitoring. Due to ongoing hematuria and symptomatic anemia, the patient received transfusions of seven units of packed red blood cells and one unit of fresh frozen plasma. Despite these measures, the clinical condition worsened. Inflammatory markers increased markedly (Table 1), consistent with a progressive systemic inflammatory response. Follow-up CT imaging confirmed persistent bilateral hydronephrosis, a necrotic-appearing bladder wall mass, and subtle perivesical fat stranding, but no evidence of free intraperitoneal air (Figures 1, 2) [4]. These findings suggested possible tumor-related wall disruption but remained non-diagnostic.

On postoperative day five following TURBT, the patient developed new symptoms consistent with acute abdomen, including diffuse abdominal pain, tense abdominal wall with guarding, and sluggish bowel sounds, suggestive of paralytic ileus. Although classical peritoneal signs were incomplete, the clinical constellation, including worsening inflammatory parameters, metabolic acidosis, and abdominal distension, raised a strong suspicion of sterile chemical peritonitis, possibly secondary to microscopic urinary leakage through necrotic tumor tissue [3]. Given the patient's rapid clinical deterioration, a multidisciplinary decision was made to perform an emergency radical cystoprostatectomy, despite pending final histopathological results from the TURBT specimen. The bladder and prostate were removed en bloc, and bilateral cutaneous ureterostomies were fashioned to achieve immediate urinary diversion.

**Figure 3:**
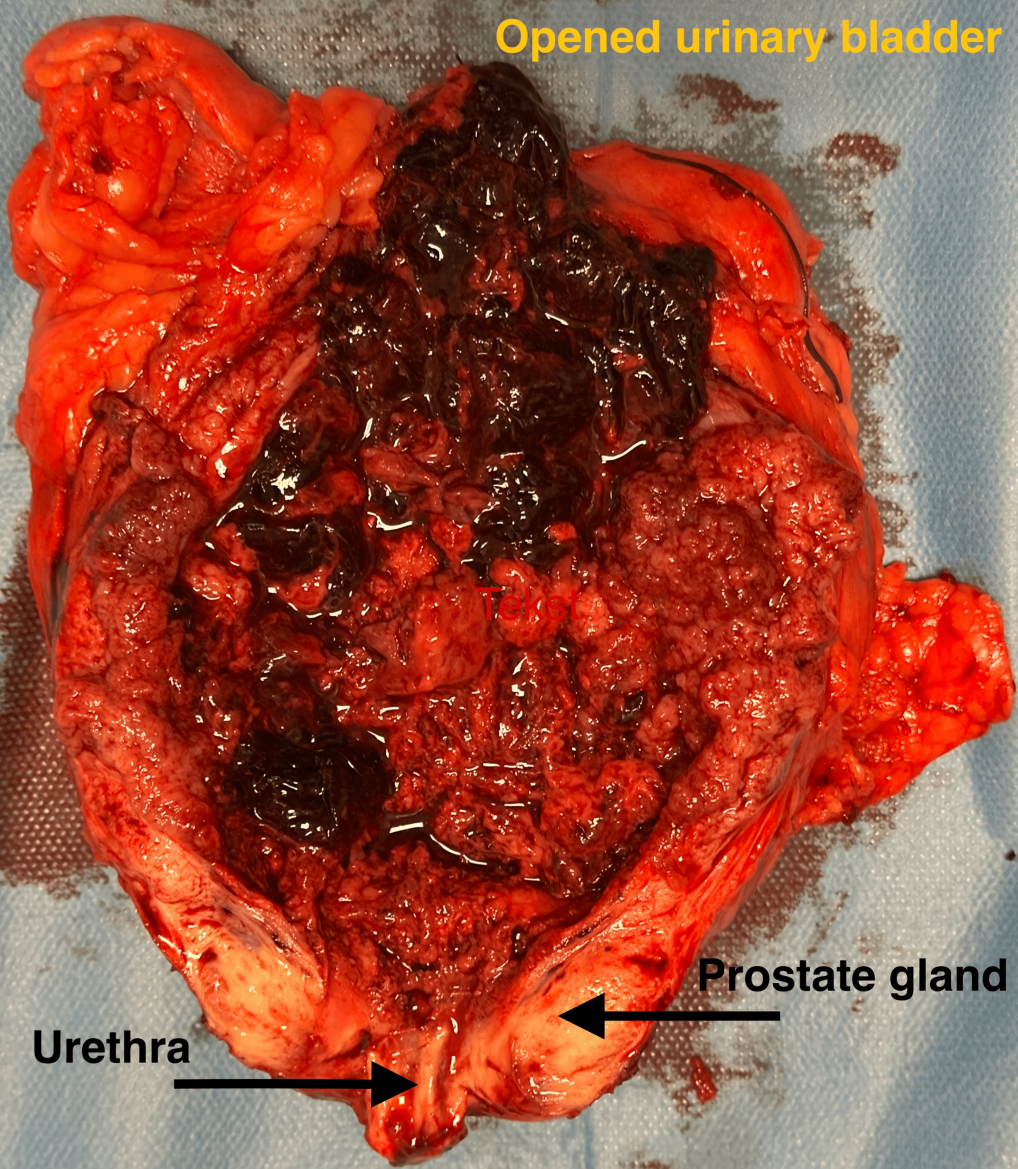
Opened urinary bladder specimen after radical cystoprostatectomy, revealing a large, friable, necrotic urothelial tumor filling the bladder cavity The urethra and prostate gland are labeled at the bottom of the image

No overt perforation of the bladder was identified during surgery. However, upon opening the resected specimen, a large, friable urothelial tumor with extensive areas of necrosis and hemorrhage was observed, occupying nearly the entire bladder cavity (Figure 3) [6]. Although macroscopic continuity of the bladder wall appeared preserved, the degree of tissue necrosis and inflammatory response raised a strong intraoperative suspicion of sterile chemical peritonitis secondary to microscopic urinary extravasation [3].

Intraoperatively collected peritoneal fluid was sterile on culture, with no bacterial growth. Cytological analysis revealed numerous inflammatory cells, but no malignant cells or organisms. Although no direct biochemical testing (e.g., creatinine concentration) was performed, the cytological and intraoperative findings were interpreted as consistent with sterile peritoneal inflammation, possibly caused by chemical irritation due to microscopic urinary leakage through necrotic tumor tissue [3].

The postoperative course was favorable. Within 48 hours, the patient showed clinical improvement, with normalization of bowel sounds, resolution of abdominal tension, decreasing inflammatory markers, improved renal function, and stabilization of hemoglobin levels without the need for further transfusions. These clinical findings were considered supportive, but not confirmatory, of the hypothesis that necrotic tumor-related urine extravasation was the trigger for the systemic inflammatory response, which resolved following radical surgery and urinary diversion [5].

**Figure 4:**
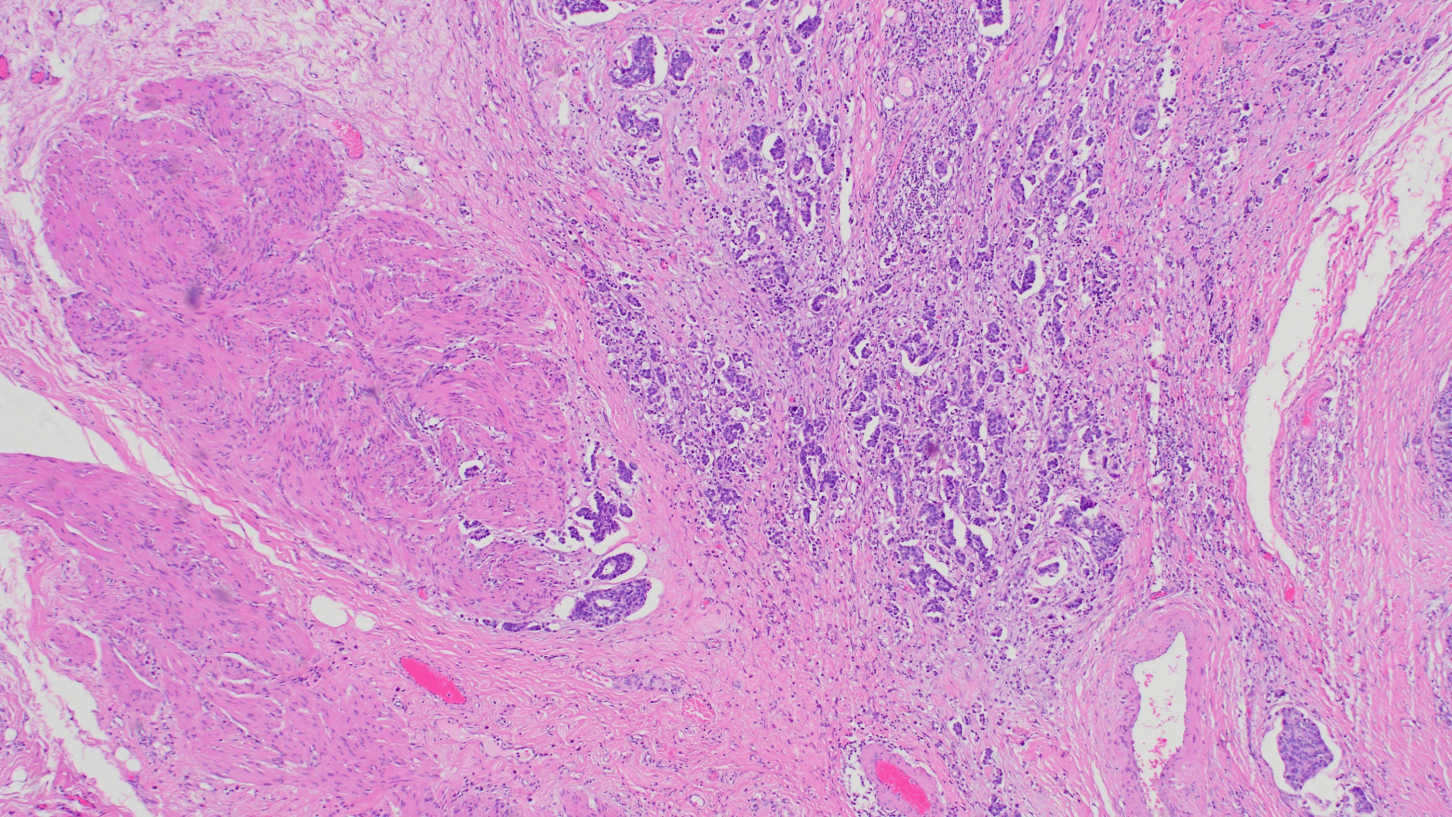
Infiltrating high-grade urothelial carcinoma with evident muscularis propria involvement The tumor invades deeply into the muscle layer (pT2b). All specimens were stained with hematoxylin and eosin (H&E), original magnification ×100

**Figure 5:**
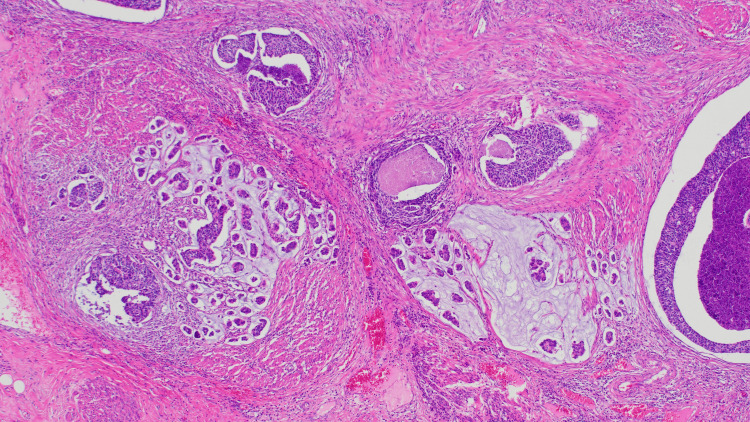
Foci of glandular differentiation and angioinvasion within the tumor stroma, supporting high-grade features and aggressiveness All specimens were stained with hematoxylin and eosin (H&E), original magnification ×100

Final histopathological examination confirmed high-grade urothelial carcinoma infiltrating the outer half of the muscularis propria (stage pT2b), as illustrated in Figure 4. Histology demonstrated marked nuclear atypia, numerous mitotic figures, and focal glandular differentiation, along with evidence of angioinvasion (Figure 5). Regional lymph nodes were not assessed (pNX).

The postoperative course remained favorable. The patient was discharged in stable condition and referred for outpatient follow-up in both urology and oncology clinics. At the 14-day follow-up visit, surgical wound healing was satisfactory, with no signs of infection or fever. Bilateral cutaneous ureterostomies were functioning properly, with unobstructed urine drainage.

## Discussion

Bladder cancer remains the most common malignancy of the urinary tract and is often diagnosed at an early, non-muscle-invasive stage [1]. However, delayed presentation or aggressive histological subtypes can result in locally advanced disease and serious complications, including hydronephrosis, bleeding, and tumor necrosis [2]. In rare cases, such as the one presented, advanced bladder tumors may be associated with peritoneal inflammation suggestive of sterile chemical peritonitis. This condition is thought to result from microscopic urinary leakage into the peritoneal cavity, potentially through tumor-infiltrated or necrotic bladder wall segments [3,4]. However, as highlighted by our case, establishing a definitive etiological mechanism remains challenging. In our patient, cytological examination of the peritoneal fluid revealed inflammatory changes without evidence of infection or malignancy, and intraoperative findings showed no overt perforation [7]. Although these findings supported the diagnosis of sterile chemical peritonitis, the absence of direct biochemical analysis (e.g., creatinine concentration in peritoneal fluid) limits diagnostic certainty.

Differential diagnosis in this clinical scenario should include several entities that can present with signs of peritoneal irritation and systemic inflammation in the context of bladder cancer. First, peritoneal carcinomatosis should be considered, particularly in advanced urothelial carcinoma. However, in our case, there were no cytological signs of malignancy in the peritoneal fluid, and the intraoperative findings lacked the classic features of peritoneal seeding or nodularity. Second, SBP may present similarly, especially in immunocompromised patients or those with chronic kidney disease. Nevertheless, negative bacterial cultures from peritoneal fluid and the absence of systemic bacteremia in this patient made SBP unlikely. Third, intestinal perforation was ruled out based on clinical examination, radiologic findings (absence of free air), and intraoperative exploration [8]. There were no signs of bowel wall compromise or contamination within the peritoneal cavity. Lastly, dialysis-associated peritonitis was not applicable, as the patient had not received peritoneal dialysis.

The overall presentation - advanced tumor with diffuse necrosis, sterile but inflammatory peritoneal fluid, and rapid improvement following surgical source control - supports the hypothesis of urine-induced chemical peritonitis, although this remains a diagnosis of exclusion. Early surgical source control appears to be crucial in cases of suspected chemical peritonitis associated with advanced bladder cancer. Although there are no formal guidelines for managing this specific scenario, urgent cystectomy in selected cases has been associated with favorable outcomes [5]. In our patient, clinical stabilization occurred rapidly after surgery, supporting the role of early and aggressive management in similar presentations.

Only a few published case reports have described comparable clinical trajectories. Maachi et al. [9] have reported a case of bladder rupture leading to peritoneal seeding and carcinomatosis, illustrating the diagnostic overlap between mechanical and neoplastic causes of peritoneal inflammation. Also, Goel and Goel [10] have described bladder carcinoma manifesting as an acute abdomen, with surgical findings mimicking bowel perforation. These cases underscore the importance of maintaining a high index of suspicion when evaluating elderly patients with advanced bladder tumors, persistent systemic inflammation, and non-specific abdominal findings. While imaging and laboratory studies can be helpful, definitive diagnosis often requires intraoperative assessment.

Ultimately, in the absence of confirmatory biochemical or cytological findings, diagnosis of sterile chemical peritonitis remains presumptive and must be interpreted in the clinical context. Further accumulation of similar case reports may help establish a more consistent diagnostic and therapeutic approach.

## Conclusions

Advanced bladder cancer may, in rare instances, be complicated by sterile chemical peritonitis due to suspected urinary extravasation through necrotic or infiltrated bladder walls. Although no overt perforation was identified, the clinical and intraoperative findings in this case were strongly suggestive of urine-induced peritoneal inflammation. Persistent systemic inflammation despite appropriate antibiotic therapy should prompt consideration of this diagnosis. Early surgical source control, including radical cystectomy and simple urinary diversion, may offer clinical benefit in rapidly deteriorating patients, especially when diagnostic uncertainty limits conservative options. However, given the absence of direct biochemical confirmation, such diagnoses remain presumptive and must be interpreted cautiously. This report emphasizes the importance of interdisciplinary collaboration and rapid decision-making in complex oncologic emergencies. Clinicians should consider this rare entity in elderly patients with unexplained peritonitis-like symptoms. Further reports and series are needed to better define diagnostic criteria and guide therapeutic strategies in similar cases.
